# Philadelphia Academy of Surgery

**Published:** 1895-02

**Authors:** 


### PHILADELPHIA ACADEMY OF SURGERY.

MEETING OF DECEMBER 3, 1894.

The PRESIDENT, DR. WILLIAM HUNT, in the chair.

DR. H. R. WHARTON exhibited

### A DUMB-BELL-SHAPED CALCULUS WHICH HAD BEEN PARTIALLY ENCYSTED, REMOVED BY LITHOTOMY.

This calculus was removed from a child five or six years of age. I show it, as it is of interest in connection with the choice of operation for the removal of calculi from the bladders of children. This patient was brought to the Children's Hospital, and on examination I found a stone in the bladder. I hesitated sometime as to what operation I should make use of. I was inclined to do litholapaxy, but when the child was etherized I made a rectal examination, and felt a prolongation of the bladder into the rectum containing the stone. I decided then upon lateral lithotomy, and after exposing the stone and attempting to grasp it, I found that it was impossible to remove it, as the posterior portion was thoroughly surrounded by the walls of the bladder. I dissected it out with my finger without breaking it. The patient after the operation did perfectly well. I believe that in a case of this kind it would be impossible to do litholapaxy, for while the projecting portion of stone could have been grasped and crushed, the encysted portion could not have been reached.

I have had very little experience with these partially encysted stones, but in two of the cases that I have recently seen I have found that a great deal can be learned by rectal examination. In the previous case I was able to make up my mind pretty clearly as to the shape and possible attachments of the stone. I show the specimen mainly for the purpose of calling attention to the difficulty of crushing stone under such circumstances. I have done litholapaxy in a child five years of age. The operation is a satisfactory one if you can completely crush the stone. In the case mentioned I used a No. 16 lithotrite, and had no trouble in crushing and removing the stone. The day after operation the urine was clear and the temperature was normal. The patient made a satisfactory recovery. Sometime ago I assisted a friend in a suprapubic lithotomy where much the same conditions were present as in the case which I have reported, the posterior portion of the stone being imbedded. In such a case the attempt to do litholapaxy would have resulted in an incomplete operation.

DISCUSSION.

DR. J. EWING MEARS: Dr. Wharton has presented a very interesting question. I think the experience of lithotomists points to the view that in children and in elderly persons the cutting is a safer one than the crushing operation, for the reason that in children, the urethra and bladder being small, there is some difficulty in the manipulation, and in elderly persons there is danger in many cases of disease of the bladder or kidneys.

It will be remembered that when Napoleon III. was operated on for stone, by crushing, and death supervened, the question was largely discussed, and it was thought if lithotomy had been performed in his case, the result might have been different.

DR. THOMAS S. K. MORTON: As bearing upon the question of the choice of operation, it may be of interest to recall an article which has recently appeared upon the subject of litholapaxy in children by Surgeon-Major Keegan. Major Keegan believes that litholapaxy is the ideal operation in children, where one can drill himself properly in the procedure, but that for those who have occasion to operate but seldom, the cutting method is far safer. His statistics, running up into hundreds of cases, show an astonishingly low mortality. Before adopting the crushing operation to children he had cut in innumerable cases. I think that this is the view that should guide us in this country, where few have an opportunity for extensive experience, as the affection is in most localities quite rare. Probably the cutting operation, either the left lateral or the suprapubic, will continue to be the safest operation for all save those who see many cases.

